# Williamsburg Asylum

**Published:** 1847-05

**Authors:** 


### WILLIAMSBURG (VA.) ASYLUM.

The annual report of the Eastern Asylum in the City of Williamsburg, Virginia, for 1846, presents a favorable view of the condition of that institution. During the year the inmates numbered one hundred and sixty. The number of discharges was thirteen, all of which, except one, were in the usual form of recoveries. Dr. Galt's report is an interesting document.

